# Identification of Immune Hub Genes in Obese Postmenopausal Women Using Microarray and Single-Cell RNA Seq Data

**DOI:** 10.3390/genes16070783

**Published:** 2025-06-30

**Authors:** Fu-Rong Zhang, Xuan Lu, Jia-Li Li, Yu-Xin Li, Wei-Wei Pang, Ning Wang, Kun Liu, Qian-Qian Zhang, Yun Deng, Qin Zeng, Xiao-Chao Qu, Xiang-Ding Chen, Hong-Wen Deng, Li-Jun Tan

**Affiliations:** 1 Laboratory of Molecular and Statistical Genetics, College of Life Sciences, Hunan Normal University, Changsha 410081, China; 202120142650@hunnu.edu.cn (F.-R.Z.); 202320142759@hunnu.edu.cn (X.L.); ljl1449257601@163.com (J.-L.L.); guanxu193@163.com (Y.-X.L.); wwpang@foxmail.com (W.-W.P.); wangning20210118@126.com (N.W.); kunl@hunnu.edu.cn (K.L.); edenw2411@163.com (Q.-Q.Z.); zengq1012@hunnu.edu.cn (Q.Z.); quxc@hunnu.edu.cn (X.-C.Q.); xdchen@hunnu.edu.cn (X.-D.C.); 2 Zebrafish Genetics Laboratory, College of Life Sciences, Hunan Normal University, Changsha 410081, China; dengyun@hunnu.edu.cn; 3 Tulane Center of Biomedical Informatics and Genomics, Deming Department of Medicine, Tulane University School of Medicine, New Orleans, LA 70112, USA; denghongwen66@yahoo.com

**Keywords:** postmenopausal women, obesity, immune hub gene

## Abstract

**Background:** Obesity is characterized by a chronic state of low-grade inflammation. Investigating immune-critical genes and their biological functions in the adipose tissue of postmenopausal obese women is crucial for elucidating the underlying mechanisms of immune dysregulation associated with obesity. **Methods:** In this study, microarray (GSE151839) and single-cell RNA-seq (GSE176171) datasets were obtained from the Gene Expression Omnibus (GEO). For microarray data analysis, weighted gene co-expression network analysis (WGCNA), protein–protein interaction network (PPI) analysis, and immune infiltration analysis (ssGSEA) were employed to identify obesity-related immune-critical genes. Subsequently, the candidate genes were validated using scRNA-seq data to explore their expression patterns at the single-cell level. Finally, the expression levels of these immune-critical genes were experimentally verified in adipose tissue from obese and control zebrafish models using RT-qPCR. **Results:** Analysis of microarray data through WGCNA, PPI and ssGSEA identified 16 obesity-related immune-critical genes, including *IL7R*, *CD3E*, *CD2*, *CCR5*, *CD3D*, *MS4A1*, *TRAT1*, *SLAMF8*, *CCL3L1*, *SPP1*, *CCL5*, *IL2RG*, *CD3G*, *TLR8*, *ITK*, and *CCL3*. Differential expression of *SPP1*, *ITK* and *CCL5* was confirmed in scRNA-seq data, with *ITK* and *CCL5* showing distinct expression patterns in natural killer (NK) cells. Furthermore, RT-qPCR analysis revealed upregulation of *SPP1* and *ITK* in adipose tissue of obese zebrafish compared to lean controls. **Conclusions:** This study identifies *SPP1*, *ITK* and *CCL5* as key immune hub genes in the adipose tissue of postmenopausal obese women, with NK cells playing a significant role in adipose tissue inflammation through the expression of these genes. These findings provide novel insights into potential therapeutic targets for the prevention and treatment of obesity in postmenopausal women.

## 1. Introduction

Obesity, characterized by the excessive accumulation of body fat, is a highly prevalent, heterogeneous, and recurrent medical condition affecting over 650 million adults globally [1]. A 2022 study by the U.S. Centers for Disease Control and Prevention (CDC) revealed that the prevalence of severe obesity is higher in women than in men across all racial and ethnic groups, with postmenopausal women being particularly susceptible to obesity and severe obesity [2,3]. The global prevalence of obesity continues to rise, contributing to a widespread public health crisis [4,5,6,7]. Obesity is also a significant risk factor for cancer; for every 5 kg/m^2^ increase in body mass index (BMI), the relative risk of developing cancer increases 1.06- to 1.62-fold [8], and individuals with a BMI exceeding 40 kg/m^2^ face a higher risk of cancer-related mortality compared to those with a normal BMI [9]. While our study focuses on obesity-related immune dysregulation, menopausal transitions also significantly impact bone metabolism [10] and cancer risk. This underscores the pleiotropic effects of hormonal changes across tissues [11]. In postmenopausal women, decreased estrogen secretion and increased glucocorticoid levels promote fat absorption and storage, disrupting lipid metabolism and predisposing this population to obesity.

Obesity is associated with a chronic state of low-grade inflammation, marked by the activation and infiltration of pro-inflammatory immune cells and the dysregulated production of pro-inflammatory cytokines. In obese individuals, adipose tissue exhibits increased release of fatty acids and hormones, reduced lipid turnover, and elevated levels of inflammatory macrophages, which drive the secretion of pro-inflammatory cytokines such as tumor necrosis factor-α (TNF-α) and interleukin-6 (IL-6) [12,13]. The aggregation of immune cells in adipose tissue alters its microenvironment, contributing to the formation of complex inflammatory networks [14]. Macrophages, key mediators of adipose tissue inflammation, are abundant in adipose tissue and can be classified into two phenotypes: M1 (pro-inflammatory) and M2 (anti-inflammatory) [15]. In lean individuals, M2 macrophages predominate, secreting anti-inflammatory cytokines such as interleukin-1 receptor antagonist (IL-1ra), interleukin-4 (IL-4), interleukin-10 (IL-10), and transforming growth factor-beta 1 (TGF-β1), which help mitigate inflammatory responses [16]. In contrast, obesity induces a shift from M2 to M1 macrophages, which secrete pro-inflammatory cytokines such as IL-1, IL-6, IL-12, TNF-α, and monocyte chemotactic protein-1 (MCP-1), and produce inducible nitric oxide synthase (iNOS). Beyond macrophages, other immune cells, including neutrophils, B-cells, mast cells, and T-cells, are also implicated in obesity-related inflammation [13].

This study aims to identify immune hub genes involved in adipose tissue inflammation in postmenopausal obese women and investigate their potential roles in immune responses. Previous studies, such as Walker et al. [17], analyzed the GSE151839 dataset (also used in this study) and identified SPP1 and SLC27A2 as the most significantly upregulated and downregulated genes in obese and normal samples, respectively. However, their work primarily focused on skin tissue and did not examine immune-specific pathways or single-cell dynamics. To address this gap, we conducted a comprehensive reanalysis of GSE151839 and an additional validation dataset (GSE176171), integrating bulk RNA-seq, single-cell RNA-seq (scRNA-seq), and experimental zebrafish models. Specifically, our study aims to: (1) identify immune hub genes in adipose tissue of postmenopausal obese women using WGCNA, PPI networks, and immune infiltration (ssGSEA); (2) validate these genes at single-cell resolution to determine their cellular origins; and (3) experimentally confirm their role in obesity-related inflammation using a zebrafish model. Our research provides a multidimensional approach linking transcriptomic insights with functional gene expression analysis.

## 2. Materials and Methods

### 2.1. Study Design

**Figure 1:**
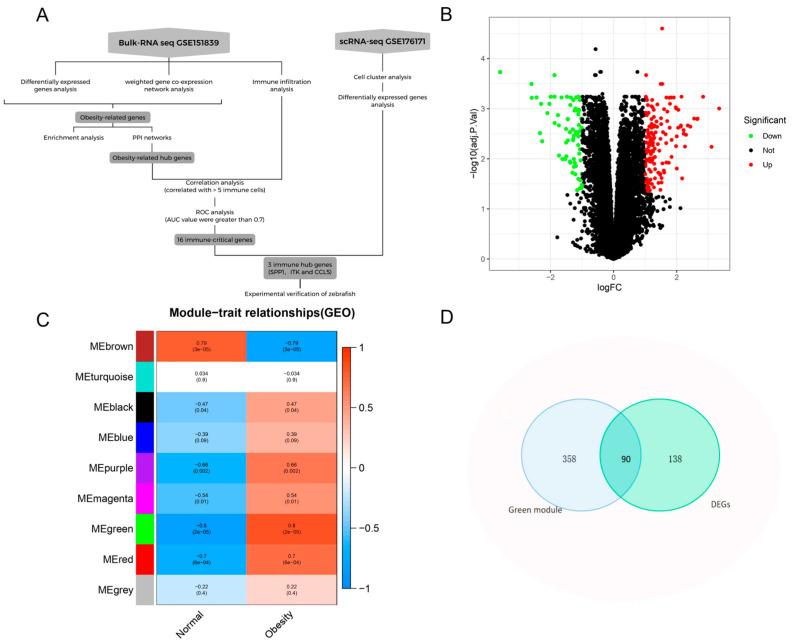
Identification of obesity-related genes. (**A**): Study flowchart. (**B**): Volcano plot of differentially expressed genes (DEGs) in adipose tissue from obese versus healthy postmenopausal women (GSE151839 microarray dataset, *n* = 10 per group). Red dots: significantly upregulated genes (log_2_FC > 1, FDR-adjusted *p* < 0.05); green dots: significantly downregulated genes (log_2_FC < −1, FDR-adjusted *p* < 0.05); black dots: non-significant genes. (**C**): Module-feature graph: Correlation between modules and obesity. (**D**): Venn diagram of the intersection genes of DEGs and green module genes.

The study design is illustrated in Figure 1A. Initially, differentially expressed genes (DEGs) analysis and weighted gene co-expression network analysis (WGCNA) were conducted on the microarray dataset. The intersection of DEGs and key module genes identified through WGCNA was selected as obesity-related genes. Functional enrichment analyses, including Gene ontology (GO), Kyoto Encyclopedia of Genes and Genomes (KEGG) and gene set enrichment analysis (GSEA), revealed that immune cells and inflammatory factors play a critical role in obesity. Subsequently, protein–protein interaction (PPI) analysis was performed on obesity-related genes to identify hub genes. Immune infiltration analysis was conducted on the microarray dataset, and Spearman correlation analysis was employed to assess the correlation between immune cells and hub genes. Through these analyses, 16 immune-critical genes were identified based on our screening criteria. Further validation was performed using single-cell RNA-seq (scRNA-seq) data to identify immune cells in obese adipose tissue and confirm the expression of three key genes (*SPP1*, *ITK*, and *CCL5*) at the single-cell level. Finally, the expression of these immune-critical genes was experimentally validated in adipose tissue using a zebrafish model.

### 2.2. Data Sources

Transcriptomic profiles were obtained from subcutaneous adipose tissue of age-matched postmenopausal women (healthy: BMI < 25; obese: BMI ≥ 30). The discovery cohort comprised microarray data (GSE151839; *n* = 10/group) [17], while the validation cohort consisted of single-cell RNA-seq data (GSE176171; *n* = 5/group) [18].

### 2.3. Data Pre-Processing

The expression matrices of both datasets were preprocessed independently. For the microarray dataset GSE151839, data filtering, log2 transformation and normalization were performed using the “limma” package (version 3.56.0) in R. Probe IDs in the expression matrix were converted to gene SYMBOL IDs using the “AnnoProbe” package (version 0.1.8). When multiple probe IDs or EMSEMBL IDs corresponded to the same gene SYMBOL, the average expression value was calculated and assigned as the gene’s expression value.

### 2.4. Analysis of Differential Gene Expression

Limma package [19] was used to screen DEGs in dataset GSE151839 with the absolute difference multiple greater than 1 (|logFC(Foldchange)| > 1) and *p* value < 0.05 after correction. A total of 228 DEGs were obtained.

### 2.5. WGCNA Analysis

WGCNA clusters highly correlated genes into modules, representing the overall expression level of each module through its signature genes, and correlates these modules with external phenotypes for further analysis. This study uses the “BioConductor” package (release 3.18) in the R programming language and the “goodSamplesGenes” function to check whether the data meets the requirements of network analysis. Pearson correlation coefficients for all genes are then calculated and the appropriate soft threshold is automatically selected through the “PickSoftThreshold” function in the WGCNA software package (version 1.72-1) [20]. The weighted gene co-expression network was constructed based on the optimal soft threshold, and modules were identified and clustered according to the similarity of gene co-expression patterns. The relationship between these modules and phenotypic traits was determined, and the minimum number of genes per module was set to 50. Gene significance (GS) values were calculated to assess the correlation between each module and obesity, and the results were visualized. The green module, which exhibited the highest correlation coefficient, was selected as the key module for further analysis.

### 2.6. Functional Enrichment Analysis of Obesity-Related Genes

DEGs and green module genes were intersected and presented using a Venn diagram. Overlapping genes were defined as obese-related genes. To further uncover the potential functions of these obesity-related genes, gene ontology (GO) enrichment analysis using the “clusterProfiler” (version 4.8.0) in the R package [21] and Kyoto Encyclopedia of Genes and Genomes (KEGG) enrichment analysis using the online tool KOBAS [22] was performed. The significance threshold for enrichment analysis was a corrected *p*-value < 0.05.

Additionally, gene set enrichment analysis (GSEA) for obesity-related genes using the pathway gene set to download from the molecular signatures database (MSigDB). pathways with a normalized enrichment score (NES) absolute value >1 and *p* adjusted <0.05 were considered statistically significant.

### 2.7. Construction of Protein–Protein Interaction Networks

PPI networks were constructed based on a string database (http://string-db.org/, accessed on March 2023) with a confidence level greater than or equal to 0.4 [23] and visualized via Cytoscape (version 3.9.0) software. CytoHubba plug-in of Cytoscape was used to identify hub genes based on maximal clique centrality (MCC). The topological analysis algorithm of MCC identified the top 29 genes in the PPI network as hub genes [24].

### 2.8. Immune Infiltration Analysis

Immune infiltration analysis of GSE151839 was performed using Single Sample Gene Set Enrichment Analysis (ssGSEA) [25]. For the ssGSEA analysis, we used TPM (Transcripts Per Million)-normalized expression values derived from the microarray dataset after preprocessing and normalization steps. TPM was chosen because it accounts for both sequencing depth and gene length, making it more suitable for between-sample comparisons, which is essential for ssGSEA where enrichment scores are calculated on a per-sample basis. We downloaded the immune infiltration gene set from http://cis.hku.hk/TISIDB/ (accessed on May 2023), which includes 28 immune cells such as macrophages and mast cells and their related genes, and performed immune infiltration analysis in this study using the GSVA software package (v1.48.0) in R. This allowed us to calculate the abundance of the 28 immune cells in each sample (including the obese group and the healthy control group). In order to reveal the relationship between hub genes and immune cells, the correlation coefficients between immune cells and the expression levels of 29 hub genes were analyzed using Spearman correlation analysis.

### 2.9. Preliminary Screening of Immunity-Related Genes

In order to further identify immune-critical genes associated with obesity, we used the following two screening criteria. Firstly, the genes should be significantly correlated with more than five immune cells (correlation coefficient > 0.73 and *p*-value < 0.05) [26]. Secondly, the genes should have diagnostic efficacy to distinguish between obesity and health. Area under the curve (auc) values were greater than 0.7 in the receiver operating characteristic curve (ROC) analysis. Only genes that meet the two criteria were preliminarily considered immune-critical genes and included for subsequent analysis.

### 2.10. Analysis of Single-Cell Datasets

The GSE176171 dataset was preprocessed by merging data from the obese and healthy groups using the “merge” function. To ensure data quality, cells with fewer than 200 or more than 10,000 expressed genes were excluded, and cells with mitochondrial gene content exceeding 30% were removed. After filtering, 33,924 cells from the healthy group and 21,783 cells from the obese group were retained for downstream analysis.

The samples were then integrated using the “CCA” function within the Seurat V5 R package to identify the top 2000 genes with high inter-cellular variation, and the top 10 genes were visualized. After that, principal component analysis (PCA) was used for linear dimensionality reduction to determine the available dimensions of the dataset, and the first 20 principal components were selected for subsequent cluster analysis. We use the Harmony R package “runHarmony” [27] function to eliminate batch effects between samples. In addition, we used the uniform manifold approximation and projection (UMAP) algorithm to reduce the dimensionality of the data. Specifically, UMAP was selected over PCA and t-SNE due to its ability to better preserve both global and local structures in high-dimensional data. Unlike PCA, which captures only linear variance, UMAP is non-linear and more suitable for capturing complex gene expression relationships. Compared to t-SNE, UMAP offers superior scalability to large datasets, faster computation, and better separation of clusters in the embedded space, which is crucial for identifying subtle immune cell subtypes and their gene expression patterns. Using the “FindClusters” function at a resolution of 0.5, we obtain 22 different clusters of cells. Next, the “FindALLMarkers” function was used to identify the upregulated genes in each cluster, and 22 cell clusters were annotated based on the marker genes [18] reported in the previous study. The distribution of immune-critical genes in each cell cluster was visualized using violin plots.

### 2.11. Zebrafish Experimental Validation

To test relevant immune marker genes in adipose tissue, we chose zebrafish as the experimental model [28,29], which is increasingly recognized as a reliable system for studying inflammation and metabolic pathways. The lipid metabolism of zebrafish was significantly similar to mammals and the occurrence of ovarian aging in zebrafish is spontaneous. Adult female zebrafish are a compelling “menopause prototype” because very low circulating levels of estradiol and testosterone are indicators that spontaneous ovarian aging occurs. The estradiol levels of older female zebrafish were significantly lower than those of younger zebrafish, suggesting a progressive decline in hormones similar to those observed in females during the transition from reproductive age to menopause [30]. This study selected adult female zebrafish to construct an obesity model to verify immune cell-related biomarkers. While acknowledging the limitations of translating zebrafish findings directly to human physiology, this model provides valuable insight into conserved biological processes.

Adult female zebrafish of the Tuebingen (TU) strain were used in this study [31]. The female TU zebrafish were divided into two dietary groups: the first group was fed with regular feeding of bumper worms as control and the second group was obesity-induced by overfeeding of the worms. The control and obese Zebrafish were kept in two tanks each with eight fish per tank during the experiment. The weight and length of anesthetized zebrafish were measured before the start (week 0) and at the end (week 8) of the feeding experiment and the body mass index (BMI) condition index was calculated. Subcutaneous adipose tissue and liver tissue were collected from control and obese groups, respectively, for real-time fluorescence quantitative PCR (RT-qPCR) analysis to verify the expression of immune-critical genes in adipose tissue.

## 3. Results

### 3.1. Identify Genes Associated with Obesity

Using limma, analysis of microarray data from subcutaneous adipose tissue (GSE151839, 10 obese vs. 10 healthy postmenopausal women) revealed 228 DEGs (limma, |logFC| > 1, FDR < 0.05). The volcano map (Figure 1B) showed 152 upregulated genes and 76 downregulated genes. The most significantly upregulated gene was SPP1 (logFC = 3.34), while the most significantly downregulated gene was SLC27A2 (logFC = −3.59).

Subsequent analysis using WGCNA was conducted. A soft threshold of 9 was selected for network construction, based on an R^2^ value of 0.9 and an average connectivity approaching 0 (Figure S1A). Module–trait correlation analysis (Figure 1C) revealed that the green, brown, and red modules were the three most significantly associated with obesity. Among these, the green module exhibited the strongest correlation and the lowest *p*-value (r = 0.81, *p* = 0.00002), thus being designated as the key module most closely associated with obesity (Figure S1B). A total of 90 intersection genes between DEGs and the green module genes were found (Figure 1D), which were subsequently classified as obesity-related genes.

### 3.2. GO and KEGG Enrichment Analysis of Obesity-Related Genes

**Figure 2:**
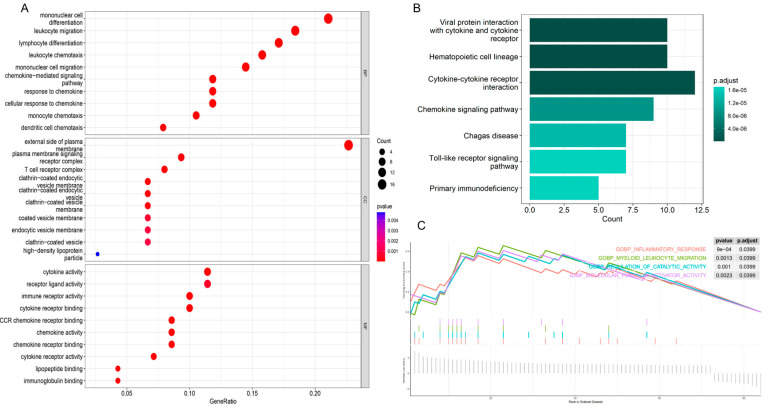
Enrichment analysis of obesity-related genes. (**A**): GO enrichment analysis of obesity-related genes. It is presented in three parts: biological process, cell component and molecular function. (**B**): KEGG enrichment analysis of obesity-related genes. (**C**): GSEA enrichment of obesity-related genes.

To elucidate the potential biological processes and pathways associated with obesity-related genes, gene ontology (GO) and Kyoto Encyclopedia of Genes and Genomes (KEGG) enrichment analyses were performed. The GO enrichment analysis (Figure 2A) revealed significant enrichment in biological processes such as “monocyte differentiation and migration”, “leukocyte migration and chemotaxis”, “lymphocyte differentiation”, and “chemokine-mediated signaling pathway”. GO enrichment in cellular components includes “outer plasma membrane”, “plasma membrane signaling receptor complex”, and “T cell receptor complex” and GO enrichment in molecular functions includes “cytokine activity”, “receptor ligand activity”, “immunoreceptor activity”, and “CCR chemokine receptor binding”.

The KEGG enrichment analysis (Figure 2B) indicated significant enrichment in pathways such as “Interaction of viral proteins with cytokines and cytokine receptors”, “T-cell receptor signaling pathway”, “Toll-like receptor signaling pathway”, “Th1 and Th2 cell differentiation”, and “Chemokine signaling pathway”.

Additionally, gene set enrichment analysis (GSEA) (Figure 2C) highlighted enrichment in pathways related to inflammatory response, myeloid leukocyte migration, regulation of catalytic activity, and molecular function activator activity. These findings suggest a pivotal role of immune cells and inflammation in the pathogenesis of obesity.

### 3.3. PPI Network Identifies Obesity-Related Hub Genes

**Figure 3:**
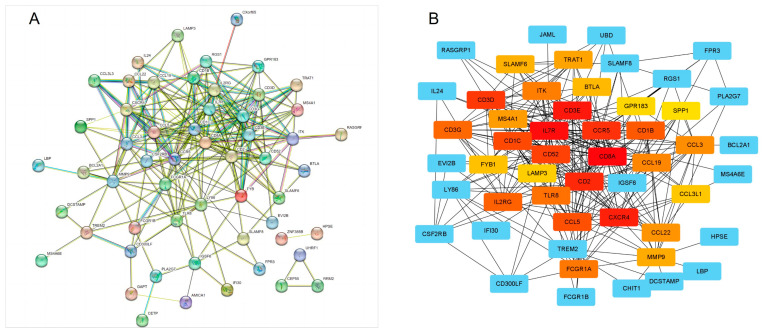
PPI network of obesity-related genes. (**A**): PPI network of obesity-related genes constructed using STRING database. (**B**): 29 hub genes were identified by MCC topological analysis algorithms using the CytoHubba plug-in (The node color is proportional to the MCC score).

To further identify obesity-related hub genes, a protein–protein interaction network was constructed using the STRING database (Figure 3A). For hub gene identification, we employed the CytoHubba plug-in in Cytoscape using the MCC algorithm, which evaluates node importance based on their participation in fully connected subnetworks. This method was chosen for its superior performance in identifying functionally essential genes compared to other topological measures. The top 29 genes ranked by MCC scores were selected as candidate hubs, representing the most interconnected nodes in the PPI network (Figure 3B). Notably, all of the hub genes identified were highly expressed in the adipose tissue of obese women, and these genes were further used for follow-up analysis.

### 3.4. Hub Gene-Immune Cell Correlation Analysis

**Figure 4:**
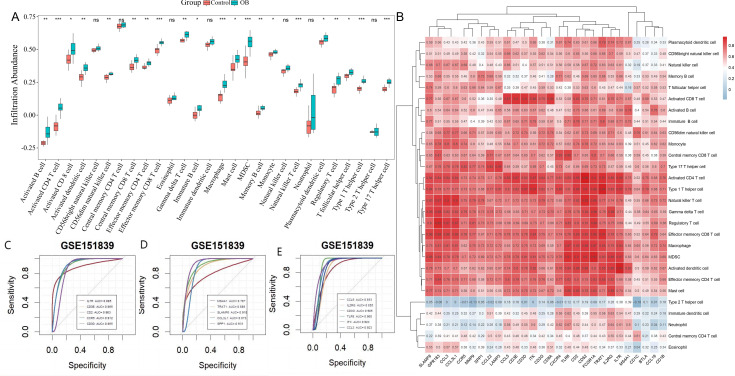
Immune infiltration analysis. (**A**): Results of immune infiltration analysis of microarray dataset. (**B**): Correlation heatmap between obesity-related hub genes and immune cell infiltration. The 16 immune-critical genes (*IL7R*, *CD3E*, *CD2*, *CCR5*, *CD3D*, *MS4A1*, *TRAT1*, *SLAMF8*, *CCL3L1*, *SPP1*, *CCL5*, *IL2RG*, *CD3G*, *TLR8*, *ITK*, and *CCL3*) are highlighted with red boxes. Color scale indicates Spearman’s correlation coefficients (blue: negative; red: positive). (**C**–**E**): Screening of immune-critical genes based on ROC values. Note: Difference in expression of 28 immune cells between obese and healthy groups. * *p* < 0.05, ** *p* < 0.01, *** *p* < 0.001.

Given the association of obesity-related genes with immune response and inflammation, immune infiltration analysis was conducted using the ssGSEA algorithm on the GSE151839 dataset. The results (Figure 4A) demonstrated that, compared to normal samples, the infiltration levels of 21 out of 28 immune cells were significantly elevated in obese samples, underscoring the importance of local and systemic inflammation in obesity development.

The correlation analysis between hub genes and immune cells (Figure 4B) showed that 22 hub genes were significantly correlated with more than 5 immune cells, of which *CD52* correlated most strongly with activated CD4 T cells (r = 0.96).

ROC analysis was performed to assess the diagnostic potential of these 22 genes. The results showed that a total of 16 genes had an AUC value greater than 0.7, indicating that these genes had a good ability to discriminate between the obese and healthy groups (Figure 4D,E). These 16 genes (*IL7R*, *CD3E*, *CD2*, *CCR5*, *CD3D*, *MS4A1*, *TRAT1*, *SLAMF8*, *CCL3L1*, *SPP1*, *CCL5*, *IL2RG*, *CD3G*, *TLR8*, *ITK*, *CCL3*) were thus initially identified as immune-critical genes associated with obesity and used for subsequent analysis.

### 3.5. Validation of Immune-Critical Genes in scRNA-Seq Data

To further validate the immune-critical genes in scRNA-seq data, the expression percentages of the 16 immune-critical genes across various cell clusters were visualized using violin plots (Figure S2). The *CCL3L1* gene, which was not expressed in any cells, was excluded. The *MS4A1* gene was expressed predominantly in B-cells, while *TRAT1* was primarily expressed in T-cells.

**Figure 5:**
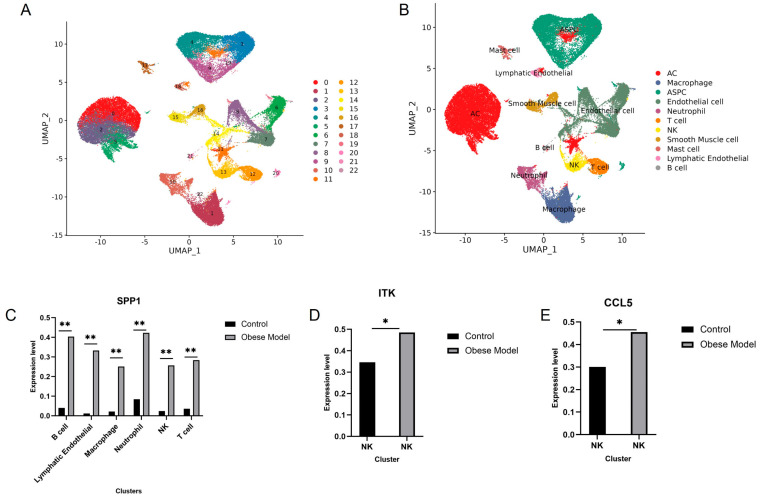
Single-cell RNA sequencing analysis of adipose tissue immune populations. (**A**): UMAP visualization of 22 cell clusters from integrated scRNA-seq data (GSE176171, *n* = 5 obese/5 lean postmenopausal women). Cells colored by unsupervised clustering (resolution = 0.5); (**B**): Cell type annotation based on canonical markers: T cells (CD3D/CD3E), B cells (MS4A1), macrophages (CD68/ADGRE1), NK cells (NCAM1/KLRD1), endothelial cells (PECAM1), and adipocyte progenitors (PDGFRA). (**C**–**E**): A bar chart of immune hub genes. * *p* < 0.05, ** *p* < 0.01.

To determine the differential expression of the 15 immune-critical genes between obese and healthy groups, a |log2FC| > 0.5 and *p*_val_adj < 0.01 were used as screening criteria. As shown in Figure 5C–E, in obese patients, *SPP1* was significantly upregulated in B cells, lymphatic endothelial cells, macrophages, neutrophils, NK cells and T cells of the obesity group, while *ITK* and *CCL5* genes were only significantly upregulated in NK cells of obesity group, which was consistent with the previous analysis results. The *SPP1*, *ITK* and *CCL5* were finally identified as immune hub genes and used for subsequent zebrafish model validation.

### 3.6. Validation of Immune Hub Genes

**Figure 6:**
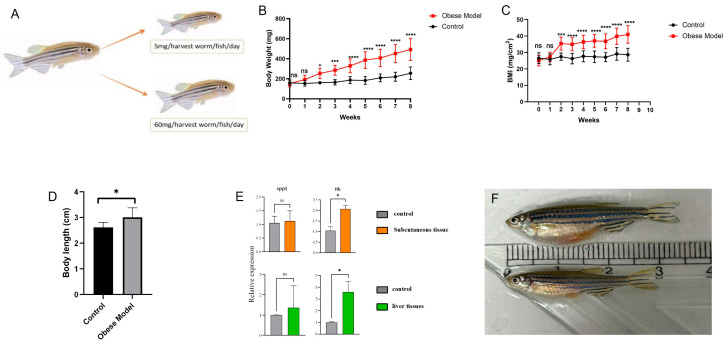
Verification of immune hub genes in zebrafish. (**A**): Overview of feeding zebrafish obesity model. (**B**): Weight changes of zebrafish during the 8-week feeding experiment. (**C**): Changes in BMI during feeding. (**D**): Changes in body length after 8 weeks of feeding. (**E**): qRT-PCR results of *SPP1* and *ITK* genes; (**F**): Side view of normal diet and overfeeding zebrafish after 8 weeks of feeding. Note: * *p* < 0.05, *** *p* < 0.001, **** *p* < 0.0001.

In microarray and scRNA-seq datasets, *SPP1*, *ITK* and *CCL5* were all highly expressed in adipose tissue of postmenopausal obese women. Consistent with the results of postmenopausal obese women, qRT-PCR validated the significantly higher expression of *SPP1* and *ITK* in the subcutaneous tissue of obese zebrafish than that of controls (Figure 6E). The expression of *ITK* was also significantly higher in the liver tissue of obese zebrafish than that of controls (Figure 6E). Since there was no homologous gene of the *CCL5* gene in zebrafish, we did not detect it.

## 4. Discussion

Obesity exhibits a higher prevalence among women compared to men, particularly in middle-aged and older populations [3]. Previous studies have also shown that obesity is associated with the frequent occurrence of menopausal symptoms [32]. In this study, we explored the composition of immune cells and identified three immune hub genes (*SPP1*, *ITK* and *CCL5*) in postmenopausal obese women based on microarray and scRNA-seq, and successfully verified the differential expression of *SPP1* and *ITK* genes through zebrafish experiments.

The identified immune hub genes, *SPP1*, *ITK* and *CCL5,* were upregulated in obese adipose tissue. *SPP1* (secreted phosphoprotein-1), a multifunctional protein implicated in various physiological and pathological processes, is highly expressed in numerous cancer types, with its expression levels positively correlated with the infiltration of dendritic cells, neutrophils, and macrophages [33]. *SPP1* is also actively expressed and secreted in macrophages at the site of inflammation, and plays an important role in cell-mediated immunity. *Tardelli* et al. discussed the potential effects of *SPP1* on the viability, cell proliferation and apoptosis of human peripheral blood mononuclear cells and macrophages [34]. Comparative studies between wild-type and *SPP1* knockout mice revealed that *SPP1* significantly enhances the survival rate of human monocytes and reduces apoptosis [35]. In addition, the proliferation of macrophages was detected in the adipose tissue of obese mice, but this proliferation was virtually absent in *SPP1* knockout mice [34]. These findings underscore the responsiveness of monocytes and macrophages to *SPP1*, their migration to inflammatory sites, and their enhanced survival and proliferation in the presence of *SPP1*. Consequently, *SPP1* emerges as a pivotal factor in adipose tissue inflammation, driving monocyte chemotaxis, macrophage differentiation, and local macrophage proliferation in obese individuals. Additionally, *SPP1* is recognized as a highly upregulated inflammatory cytokine in obese adipose tissue (33), aligning with the results of this study. Recent studies also suggest that obesity can be prevented and reversed by inhibiting the aromatics receptor to reduce the liver expression of the *SPP1* target gene [36]. Notably, the hub gene *SPP1* (osteopontin) identified here is also implicated in postmenopausal osteoporosis [37], suggesting shared pathways between metabolic and skeletal disorders.

*ITK* and *CCL5* exhibited significant differential expression specifically in natural killer (NK) cells. NK cells, a subset of lymphocytes bridging the innate immune system, are capable of eliminating infected or transformed cells without prior activation [38]. The main mechanism by which NK cells kill target cells is the secretion of prefabricated particles containing perforin and granase, which together induce apoptosis of target cells. After direct contact with target cell receptors, NK cells can also activate apoptosis by binding to the death receptor pathway. In addition, NK cells are also rapid producers of inflammatory cytokines such as IFN-γ and TNF-α, which promote the cytotoxicity of NK cells [39,40]. In an earlier 2017 study, Viel and colleagues reported increased expression of activating markers such as CD69 on NK cells in obese patients, while loss of NK cell function could be measured by degranulation and production of macrophage inflammatory protein (MIP) -1β or IFN-γ [41]. They produced less IFN-γ, had lower expression levels of granzyme B and perforin, and were less cytotoxic to tumor target cells. Recent studies have linked this NK cell dysfunction to conditions in which cell metabolism is impaired. NK cells from obese mice or obese humans cannot perform metabolic responses when stimulated by cytokines, and their metabolic rates are significantly reduced compared to NK cells from lean mice or humans [42]. This metabolic dysfunction is associated with lipid accumulation in NK cells driven by peroxisome proliferator activation receptors, resulting in altered gene expression, downregulation of mTORC1 (signal transduction complex) signaling, and decreased glycolysis and oxidative phosphorylation ratios [43].

In human NK cells, *ITK* induced by tyrosine kinase IL-2 can differentially regulate different NK-activated receptors, thus playing different roles in different pathways regulating cell-mediated cytotoxicity [44]. In mice that began high-fat feeding, NK cells lost their ability to kill macrophages and increased IFN-γ production, which boosted the recruitment of inflammatory macrophages and promoted obesity-related metabolic deficits [45]. In addition, *ITK* has also been shown to be critical for the production of Th2 and Th17 cells, and it has been found that inhibiting the kinase activity of *ITK* will lead to a significant reduction in the ability of Th1, Th2, and Th17 cells to produce IFNγ, IL-4, or IL-17. Taken together, these studies provide strong evidence that NK cells are involved in the initiation of macrophage-driven inflammatory phenotypes in obese adipose tissue [46]. In summary, in human NK cells, *ITK* plays a different role in regulating functions initiated by different activation receptors. Regulation of NK cell function by targeting *ITK* is associated not only with improved tumor immune surveillance and viral immunity, but also with reduced severity of autoimmune diseases [47].

In recent years, chemokines and chemokine receptors have become increasingly important in adipose tissue macrophage recruitment and insulin resistance associated with obesity. *CCL5* is a chemokine that recruits white blood cells to inflammatory tissue [48]. The previous study shows that *CCL5* gene expression is elevated in adipose tissue of obese mice and is associated with inflammation and the progression of insulin resistance in obese humans and mice [49], which is consistent with the results of this study. For example, mice with tissue-derived *CCL5* deficiency were protected against adipose tissue inflammation and insulin resistance induced by a high-fat diet. Blocking CCL5/CCR5 signaling reduces body weight in obese mice and prevents hyperlipidemia-induced inflammatory atherosclerotic remodeling [50]. In addition, the mRNA expression level of *CCL5* was positively correlated with the macrophage marker *CD11b* and the T cell marker *CD3* in the visceral adipose tissue of obese patients [51]. *CCL5* regulates a variety of immune cells, including the transport of T cells, monocytes, eosinophilia, etc., which can bind to at least four receptors, including *CCR1*, *CCR3*, *CCR5* and *GPR75*, among which *CCR5* is the main receptor of *CCL5* in adipose tissue. Studies have shown that obesity-induced adipose tissue enhances CCL5/CCR5 signal transduction [50]. However, the main cellular source of *CCL5* in obese adipose tissue remains unclear. In this study, *CCL5* was significantly upregulated only in NK cells, and *CCL5* was positively correlated with NK cells in the immune infiltration results of the microarray dataset (r = 0.66). Therefore, combined with the results of this study, it is suggested that NK cells are the main cell source leading to upregulation of *CCL5* in adipose tissue of postmenopausal obese women.

The identification of *SPP1, ITK,* and *CCL5* as immune hub genes in the adipose tissue of postmenopausal obese women provides new insights into the immune dysregulation associated with obesity. NK cells, known for their role in immune surveillance, appear to be key players in the inflammatory response in adipose tissue. Previous studies established that obesity-associated adipose inflammation is primarily driven by macrophage polarization (M1-type) and TNF-α/IL-1β overexpression [52], with CD8+ T-cell infiltration as an early trigger [53]. Our menopausal-specific cohort reveals a distinct NK-cell-centric mechanism, characterized by selective upregulation of *ITK* and *CCL5* (Figure 5D,E). While *SPP1* has been implicated in general obesity [54], its markedly higher expression in postmenopausal women and pro-inflammatory effects in zebrafish (Figure 6E) suggest estrogen loss amplifies its pathogenicity. However, our findings remain associative, and further studies using gene knockdown/overexpression models in both zebrafish and human tissues are needed to definitively establish causal relationships.

While our study integrated multiple layers of evidence, we acknowledge several limitations. First, the limited clinical information available in public databases and the small sample sizes of some datasets may introduce potential bias and variability. This limitation may affect the statistical power and generalizability of our findings. However, we mitigated this by integrating multiple layers of evidence (co-expression networks, immune infiltration analysis, and scRNA validation) to strengthen our conclusions. Second, while we used zebrafish as an in vivo model to validate expression patterns and test the broader immune response, the role of *CCL5* in this context may not be fully replicated in zebrafish. *CCL5*’s role in human adipose inflammation remains speculative based on our current findings, and further studies using human or other mammalian models are needed to directly validate the function of *CCL5* in this context. Additionally, we did not directly measure confounding factors such as hormone levels, dietary intake, or physical activity, all of which could influence immune responses in adipose tissue. Future studies should incorporate these variables to better understand the factors driving inflammation in postmenopausal obesity. Finally, elucidating the roles of immune hub genes in obese adipose tissue through gene knockdown experiments is essential. In summary, this study employed bioinformatics analysis of microarray and scRNA-seq datasets to identify three immune hub genes—*SPP1*, *ITK*, and *CCL5*—associated with obesity. The findings suggest that NK cells are the primary source of *CCL5* upregulation in the adipose tissue of postmenopausal obese women. Investigating the immune microenvironment in obesity and identifying immune hub genes may provide potential therapeutic targets for more effective treatments for this population.

## Data Availability

The data that support the findings of this study are openly available in Gene Expression Omnibus (GEO) database at doi.org/[10.1038/s41598-020-70244-2 & 0.1038/s41586-022-04518-2], reference number [GSE151839 and GSE176171].

## Supplementary Materials

The following supporting information can be downloaded at: https://www.mdpi.com/article/10.3390/genes16070783/s1, Figure S1: WGCNA. A: Set the soft threshold based on R^2^ = 0.9. B: The correlation between module membership and obesity gene significance in green module. Figure S2: Expression patterns of candidate immune-critical genes across cell clusters. Violin plots show the distribution of initially identified immune-related genes in annotated cell types from scRNA-seq analysis (GSE176171).
