# Supplementary material for: Identification of Immune Hub Genes in Obese Postmenopausal Women Using Microarray and Single-Cell RNA Seq Data
Source: Genes (Basel). 2025 Jun 30;16(7):783. doi: 10.3390/genes16070783 (PMC12294730; doi:10.3390/genes16070783)
Supplement: Supplementary file 1 [file genes-16-00783-s001.zip › genes-3658137-supplementary.pdf]

Figures legends

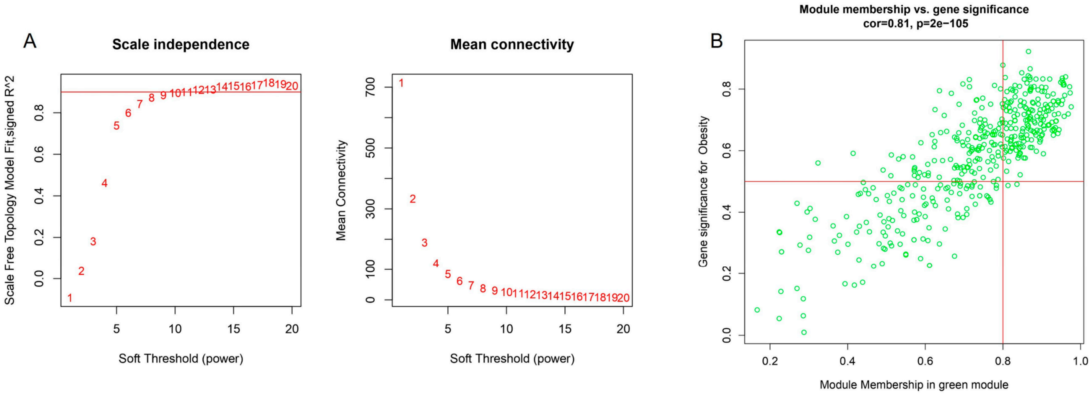

Figure S1. WGCNA. A: Set the soft threshold based on R^2=0.9. B: The correlation between module membership and obesity gene significance in green module.

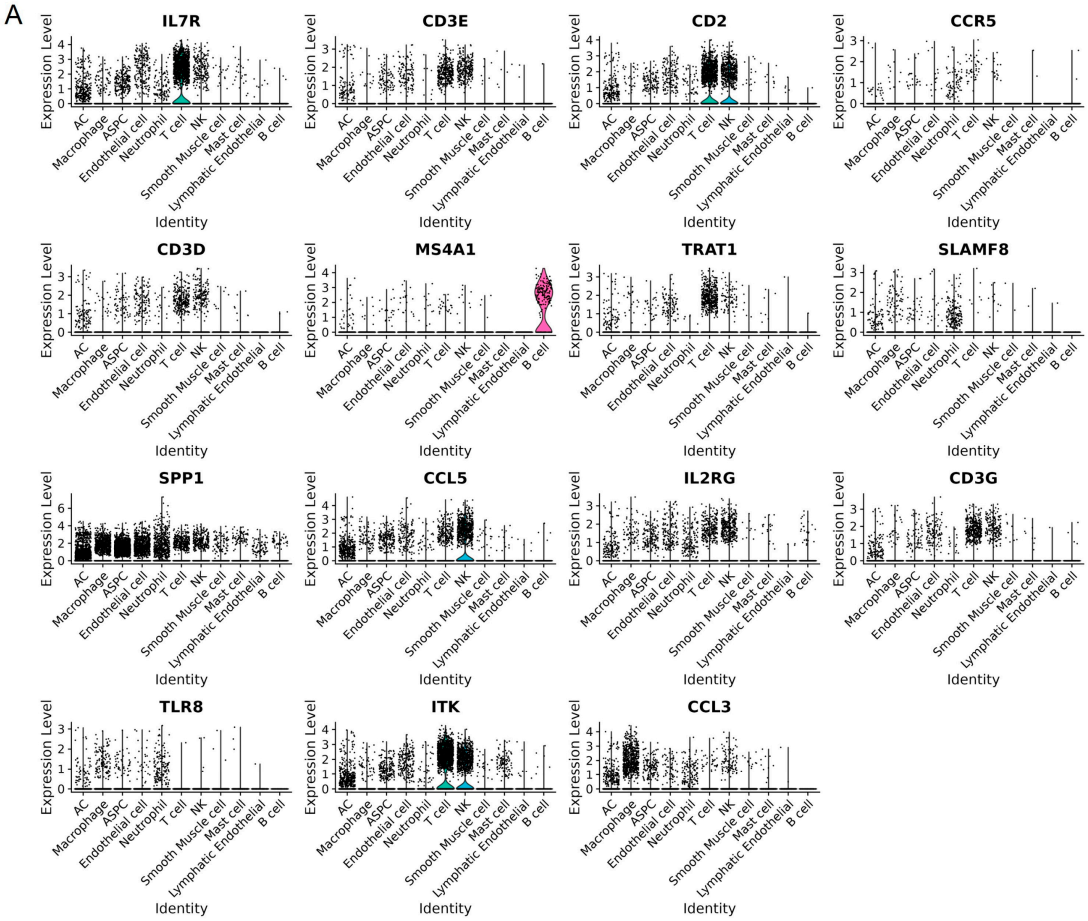

Figure S2. Expression patterns of candidate immune-critical genes across cell clusters. Violin plots show the distribution of initially identified immune-related genes in annotated cell types from scRNA-seq analysis (GSE176171).
